# Effect of central lesions on a spinal circuit facilitating human wrist flexors

**DOI:** 10.1038/s41598-018-33012-x

**Published:** 2018-10-04

**Authors:** Stefane A. Aguiar, Supriyo Choudhury, Hrishikesh Kumar, Monica A. Perez, Stuart N. Baker

**Affiliations:** 1 Institute of Neuroscience, Newcastle University, Newcastle Upon Tyne, United Kingdom; 2 Department of Neurology, Institute of Neurosciences, Kolkata, India; 3 Department of Neurological Surgery, The Miami Project to Cure Paralysis, Miami, United States

## Abstract

A putative spinal circuit with convergent inputs facilitating human wrist flexors has been recently described. This study investigated how central nervous system lesions may affect this pathway. We measured the flexor carpi radialis H-reflex conditioned with stimulation above motor threshold to the extensor carpi radialis at different intervals in fifteen patients with stroke and nine with spinal cord injury. Measurements after stroke revealed a prolonged facilitation of the H-reflex, which replaced the later suppression seen in healthy subjects at longer intervals (30–60 ms). Measurements in patients with incomplete spinal cord injury at cervical level revealed heterogeneous responses. Results from patients with stroke could represent either an excessive facilitation or a loss of inhibition, which may reflect the development of spasticity. Spinal cord injury results possibly reflect damage to the segmental interneuron pathways. We report a straightforward method to assess changes to spinal circuits controlling wrist flexors after central nervous system lesion.

## Introduction

A number of non-invasive techniques have been described in the literature to assess the function of different spinal circuits in humans – including pre-synaptic inhibition and reciprocal inhibition, amongst others^1–3^ – representing an important contribution to the understanding of human spinal circuitry. Recently, a novel spinal circuit has been described in which both wrist flexor afferents and wrist extensor Ib afferents (from Golgi tendon organs) facilitate wrist flexors in healthy humans. This circuit can be easily assessed non-invasively by conditioning the flexor carpi radialis (FCR) H-reflex with stimulation above motor threshold (MT) of the extensor carpi radialis (ECR). At an interval of 30 ms between stimuli, a clear facilitation is observed as a consequence of convergent excitatory inputs to the wrist flexor. A later inhibition at 70 ms interval is also observed, but the pathways involved are still unclear. Although the description of the circuit in healthy subjects was important, it is still unknown how central nervous system lesions may be related to the circuit and could affect the observed results of the assessment. The purpose of this study was to explore how central lesions may affect this spinal circuit.

In this study we tested the spinal circuit described by Aguiar and Baker^4^, with converging input from flexor afferents and extensor Ib afferents to the wrist flexor, in patients with stroke and spinal cord injury to investigate the effect of central lesions to this circuit. We found a prolonged facilitation of the flexor H-reflex at long intervals in subjects after stroke, which could reflect either excessive facilitation or loss of inhibition and heterogenous responses after spinal cord injury possibly reflecting damage to segmental interneuron pathways.

## Methods

Fifteen patients who had suffered a stroke, 32 to 73 years of age (ten male and five female), and nine individuals with spinal cord injury, 28 to 73 years of age (eight male and one female), participated in this study. All procedures in stroke patients were approved by the Ethical Board of the Institute of Neurosciences, Kolkata, India, where these were conducted, and all procedures in spinal cord injury patients were approved by the Ethical Committee of the University of Miami, where these were conducted. Results in patients were compared with our previously published data^4^ in 17 healthy subjects, gathered at Newcastle University and approved by the Ethics Committee of the Newcastle University Medical Faculty. All methods were carried out in accordance with the relevant guidelines and regulations of the institutions where the work was conducted. Written informed consent was obtained from all subjects prior to participation. The Ashworth scale was used to quantify the level of spasticity in individuals with stroke and spinal cord injury. Patients with stroke were classified within spasticity levels 0 (n = 2), 1 (n = 3), 1 + (n = 3), 2 (n = 6) and 3 (n = 1), and represented a mixture of hemorrhagic and Ischaemic strokes; time after stroke varied from 3 months to 10 years. The arm tested in these patients was the one corresponding to the most affected side of the body. Individuals with spinal cord injury were classified within spasticity levels 0 (n = 3), 1 (n = 1), 2 (n = 3) and 3 (n = 2); time post-lesion and level of damage varied from 1 to 32 years and from C3 to C7, respectively. Two out of 9 individuals with SCI were categorized by the American Spinal Cord Injury Impairment Scale (AIS) as AIS A (complete injury) due to the lack of sacral sparing^5^, despite being able to elicit voluntary force with wrist muscles. The right arm was tested for all spinal cord injury patients; eight out of nine of these subjects had been right handed by self-report prior to their injury. Table 1 presents general information about both groups of patients.

**Table 1:** General information about patients with stroke and spinal cord injury and their lesions.

Group	Patient	Gender	Age (years)	Time since lesion (months)	Spasticity level (Ashworth Scale)	Type of Stroke	Location of Lesion
Stroke	PSB	Male	64	11	0	Ischaemic	Cortico-subcortical
	SPS	Male	34	19	1	Ischaemic	Cortical
	SG	Male	51	125	1+	Hemorrhagic+ Ischaemic	Subcortical
	NS	Male	69	36	2	Hemorrhagic+ Ischaemic	Cortico-subcortical
	NRN	Female	73	14	0	Hemorrhagic+ Ischaemic	Cortico-subcortical
	NC	Female	34	21	1	Hemorrhagic	Cortico-subcortical
	AK	Male	54	16	1	Hemorrhagic	Subcortical
	MP	Female	35	3	1+	Ischaemic	Cortico-subcortical
	SK	Male	54	5	1+	Hemorrhagic	Cortico-subcortical
	NKM	Male	57	26	2	Ischaemic	Cortico-subcortical
	BM	Male	44	58	2	Hemorrhagic	Cortico-subcortical
	AB	Male	68	7	2	Ischaemic	Cortico-subcortical
	SWB	Female	48	23	2	Ischaemic	Cortico-subcortical
	SPP	Male	49	21	2	Hemorrhagic	Cortico-subcortical
	ND	Female	52	10	3	Ischaemic	subcortical
						**AIS Classification**	**Level of Lesion**
Spinal cord injury	AM	Male	74	264	0	A	C4
	LM	Female	56	107	0	D	C4-C5
	HF	Male	28	97	0	A	C5-C6
	JB	Male	70	381	2	C	C5-C7
	PG	Male	42	240	1	B	C5-C6
	DB	Male	57	151	3	D	C5-C6
	PL	Male	62	96	2	D	C5-C6
	GM	Male	60	12	3	D	C5
	JH	Male	45	276	2	C	C3-C4

Patients with stroke and spinal cord injury were tested with the main protocol described for healthy humans in Aguiar and Baker^4^. Subjects were seated with their forearm placed comfortably on a table in front on them. The FCR H-reflex was recorded using surface electromyography (EMG) recording electrodes (Kendall H59P, Medcat) and a Digitimer NL 824 amplifier (gain 2000, bandpass 30 Hz-2 kHz). The active EMG electrode was placed over the FCR muscle belly, and the reference over the brachioradialis. The median nerve was stimulated just proximal to the elbow using a bipolar felt pad electrode secured with a strap (P20-4zl, Medcat; cathode proximal, pulse width 0.5 ms) connected to an isolated constant current stimulator (Digitimer DS7A). Electrode placement was according to Jabre^6^. Responses to median nerve stimulation were identified as H-reflexes if they had latencies between 12 and 25 ms, and an amplitude which increased during voluntary contraction and varied with stimulus intensity in a characteristic inverted U relationship^6^. Stimulus intensity was adjusted to produce an H-reflex with amplitude around 50% of maximum. The H-reflex was conditioned by preceding stimulation of the ECR muscle. This used adhesive electrodes identical to those used for EMG recording, placed over the ECR muscle belly (cathode proximal, 1 ms pulse width), at an intensity 3x motor threshold (MT, defined as the intensity which generated the first visible muscle twitch). Eighteen different inter-stimulus intervals (ISI) ranging from 1.5 to 1000 ms were used. One combination of test and conditioning stimuli was delivered every 4 s, with the interval chosen in pseudo-random order. Ten repetitions of each ISI and twenty repetitions for the unconditioned control H-reflex were completed.

The peak-to-peak H-reflex amplitude was measured and expressed as a percentage of the control values for each subject. Significant differences from 100% across subjects were assessed using t-tests at each interval (p < 0.05). Difference between the subject groups (healthy, stroke and spinal cord injury) were assessed using an AVOVA, with factors group and ISI, followed by pairwise t-tests at a given interval between each patient group and healthy controls.

Median nerve and ECR stimulation used intensities up to 12.7 mA and 49.8 mA, respectively, for patients with stroke and 18.8 mA and 42 mA, respectively, for patients with spinal cord injury. We also measured the correlation between spasticity level and H-reflex amplitude at 30 ms and 70 ms ISIs. The datasets generated and analysed during the current study are available from the corresponding author on reasonable request.

## Results

**Figure 1:**
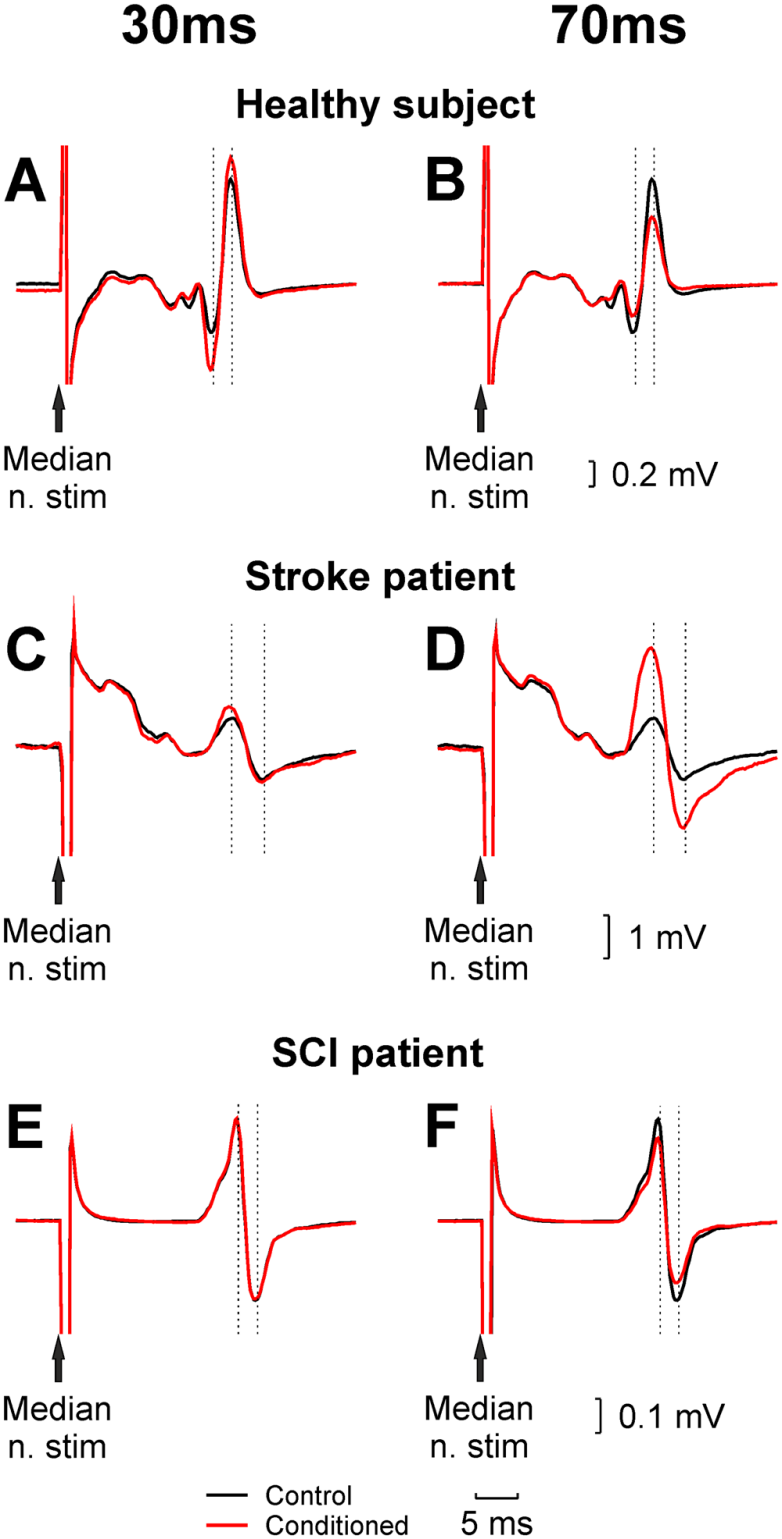
Example H-reflex recordings from the FCR muscle. Each panel shows an unconditioned H-reflex (black), and an H-reflex conditioned by preceding stimulation of the ECR muscle (red). Results are shown for a single healthy subject (**A**,**B**) patient with stroke (**C**,**D** Ashworth score 2) and spinal cord injury (**E**,**F** Ashworth score 0). Left panels (**A**,**C**,**E**) used a conditioning interval of 30 ms, which generates facilitation in healthy subjects; right panels (**B**,**D**,**F**) used an interval of 70 ms, at which the reflex is suppressed in healthy subjects.

Figure 1 shows raw reflex traces from single subjects, which illustrate the main findings of our study. As we have reported previously in healthy subjects^4^, when the median nerve stimulation was preceded by ECR stimulation at a 30 ms interval, a robust facilitation of the H-reflex was observed (Fig. 1A). By contrast, at a 70 ms interval, the H-reflex was suppressed (Fig. 1B). For the individual with stroke illustrated in Fig. 1C, the reflex was also facilitated at a 30 ms ISI, similar to the healthy subject. However, this facilitation persisted for longer intervals, so that at a 70 ms ISI the reflex was larger than the control measurement (Fig. 1D). In the single subject with spinal cord injury shown (Fig. 1E,F), there were no significant changes in the reflex amplitude by prior conditioning by ECR stimulation at either 30 ms or 70 ms interval.

**Figure 2:**
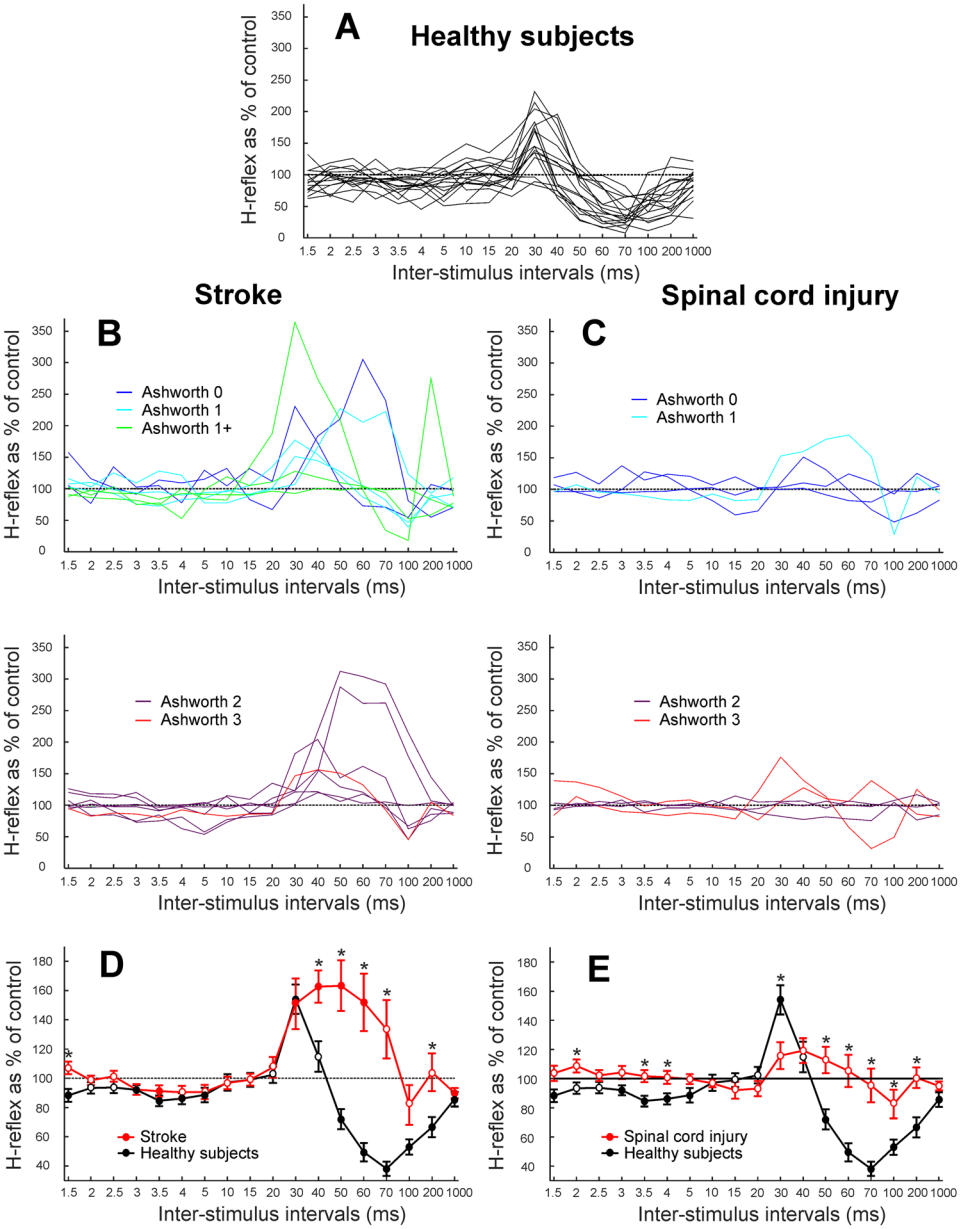
Measurement of H-reflex amplitude when conditioned by ECR muscle stimulation, as a percentage of the unconditioned reflex. Values are plotted against the inter-stimulus interval. (**A)** Overlain individual results from 17 healthy volunteers. (**B**) Overlain results from 15 patients with stroke. (**C**) Overlain results from 9 patients with spinal cord injury. In (**B**,**C**) plots have been separated according to the level of spasticity, determined by the Ashworth scale. (**D**) Average data from all individuals with stroke (red). (**E**) Average data from all individuals with spinal cord injury (red). In (**D**,**E**) the average from healthy subjects (black) is shown for comparison. Filled circles represent responses significantly different from unconditioned reflex (paired t-test, p < 0.05) and error bars represent standard error. Asterisks mark intervals with significant differences between the patient group and healthy controls (p < 0.05, t-test).

Figure 2 shows how the reflex amplitude as a percentage of the unconditioned reflex varied with ISI. Each plot presents the results for an individual subject, for healthy subjects (Fig. 2A), patients with stroke (Fig. 2B), and spinal cord injury (Fig. 2C). For the patients, traces have been split into different graphs depending on the level of spasticity measured by the Ashworth scale. Figure 2D,E show average results across patients with stroke and spinal cord injury respectively (red lines), with data from healthy subjects overlain in black for comparison. On these plots, filled symbols indicate points where the conditioned H-reflex was significantly different from the unconditioned reflex (100%) using a paired t-test (p < 0.05). An ANOVA indicated a significant effect of group (healthy, stroke, spinal cord injury; p < 10^−20^), ISI (p < 10^−19^) and their interaction (p < 10^−18^). Pairwise t-tests between each patient group and the healthy controls indicated significant differences at the intervals indicated with asterisks on Fig. 2D,E (p < 0.05).

In subjects with stroke (Fig. 2B,D), a similar facilitation at 30 ms was seen as previously described for healthy subjects^4^, but this was greatly prolonged, and the suppression normally seen in healthy adults at later intervals was lost. The level of facilitation did not correlate significantly with spasticity as measured by the Ashworth scale, at ISIs of 30 or 70 ms (R = −0.19 and R = 0.03, respectively; both p > 0.05). In individuals with spinal cord injury (Fig. 2C,E), no significant facilitation or suppression was seen in the averaged results at any of the ISIs tested. This reflected the highly heterogeneous nature of the individual datasets. After spinal cord injury, the H-reflex amplitude also did not correlate significantly with spasticity as measured by the Ashworth scale, at ISIs of 30 or 70 ms (R = 0.28 and R = −0.06, respectively; both p > 0.05).

## Discussion

In this study we tested a spinal circuit mediated by converging input from FCR afferents and ECR Ib afferents to the wrist flexor in patients who had suffered a stroke or spinal cord injury to explore the effects of central lesions to the circuit. We found a prolonged facilitation of the flexor H-reflex at long intervals after stroke, reflecting either excessive facilitation or loss of inhibition. Heterogenous responses after spinal cord injury possibly reflected damage to segmental interneurons.

In patients with stroke the facilitation at 30 ms ISI was similar to healthy adults, but it was prolonged to later ISIs which normally show suppression. One possibility is that even in healthy individuals, both facilitation and suppression processes occur concurrently, with suppression normally dominating around 70 ms ISI. In this case, the abnormal switch to facilitation could represent an increase in facilitation, or a loss of inhibition. Loss of inhibition related to changes in KCC2 function has been demonstrated to contribute to spasticity after both stroke and spinal cord injury^7,8^. However, no correlation between spasticity level and H-reflex amplitude at either 30 ms or 70 ms ISI was observed, in either patients with stroke or spinal cord injury. It is possible that any correlation was too weak to be detected with the relatively small numbers of patients which we had in our cohorts, although previous work has also failed to find a correlation between spasticity in the upper limb and H-reflex parameters^9^. While the spinal circuits involved in the 30 ms facilitation have been described^4^, the pathways involved in the later inhibition at 70 ms are still unclear. Further work is required to understand this pathway and to uncover the mechanisms underlying the reported changes in stroke survivors. Understanding the abnormalities in these responses might help to understand, and possibly treat, spasticity.

In individuals with spinal cord injury highly heterogenous conditioning curve patterns were observed, including subjects with results similar to healthy humans, similar to stroke patients and subjects with no modulation of the H-reflex for any of the ISIs tested. These different curve patterns presented no evident correlation with the level of lesion. We suggest that variation in the extent of damage to spinal segmental circuitry may lead to such a high diversity, compared to the more homogenous results in stroke. One factor in this could be the side tests: in patients with stroke, we tested the most affected arm, but in spinal cord injury the right side was consistently tested, although it was not almost the most affected. Since the H-reflex changes after ECR stimulation are a spinally mediated response which are influenced by descending control^10^ is perhaps not unexpected that stroke survivors will show similar curve patterns resulting from corticospinal tract loss alone, whereas spinal cord injury leads to more diverse findings reflecting the variable damage to spinal segmental circuitry.
